# Comparing the clinical efficacy of COVID-19 vaccines: a systematic review and network meta-analysis

**DOI:** 10.1038/s41598-021-02321-z

**Published:** 2021-11-23

**Authors:** Victoria Rotshild, Bruria Hirsh-Raccah, Ian Miskin, Mordechai Muszkat, Ilan Matok

**Affiliations:** 1 Pharmacoepidemiology Research Unit, Institute for Drug Research, School of Pharmacy, Faculty of Medicine, The Hebrew University of Jerusalem, P.O.B. 12272, 9112102 Jerusalem, Israel; 2 Jerusalem Distric, Clalith Health Services Community Division, Jerusalem, Israel; 3 Department of Cardiology, Hadassah University Hospital Ein Karem, Jerusalem, Israel; 4 Department of Medicine, Hadassah University Hospital Mt. Scopus, Jerusalem, Israel

**Keywords:** Viral infection, Epidemiology

## Abstract

New Coronavirus Disease 2019 (COVID-19) vaccines are available to prevent the ongoing severe acute respiratory syndrome coronavirus 2 (SARS-CoV-2) pandemic. We compared the efficacy of new COVID-19 vaccines to prevent symptomatic and severe disease in the adult population and to prevent symptomatic COVID-19 among the elderly. Leading medical databases were searched until August 30, 2021. Published phase 3 randomized controlled trials (RCTs) evaluated efficacy of the vaccine to prevent symptomatic and sever COVID-19 in adults were included. Two reviewers independently evaluated the literature search results and independently extracted summary data. The risk of bias was evaluated using the Cochrane Risk of Bias Assessment Tool. We performed a network meta-analysis (NMA) according to PRISMA-NMA 2015 to pool indirect comparisons between different vaccines regarding their relative efficacy. The primary outcomes were the efficacy of the vaccine against symptomatic COVID-19 in adults (PROSPERO registration number: CRD42021235364). Above 200,000 adult participants from eight phase 3 RCTs were included in NMA, of whom 52% received the intervention (active COVID-19 vaccine). While each of nine vaccines was tested in the unique clinical trial as compared to control, based on indirect comparison, BNT162b2 and mRNA-1273 vaccines were ranked with the highest probability of efficacy against symptomatic COVID-19 (P-scores 0.952 and 0.843, respectively), followed by Gam-COVID-Vac (P-score 0.782), NVX-CoV23730 (P-score 0.700), CoronaVac (P-score 0.570), BN02 (P-score 0.428), WIV04 (P-score 0.327), and Ad26.COV2.S (P-score 0.198). No statistically significant difference was seen in the ability of the vaccines to prevent symptomatic disease in the elderly population. No vaccine was statistically significantly associated with a decreased risk for severe COVID-19 than other vaccines, although mRNA-1273 and Gam-COVID-Vac have the highest P-scores (0.899 and 0.816, respectively), indicating greater protection against severe disease than other vaccines. In our indirect comparison, the BNT162b2 and mRNA-1273 vaccines, which use mRNA technology, were associated with the highest efficacy to prevent symptomatic COVID-19 compared to other vaccines. This finding may have importance when deciding which vaccine to use, together with other important factors as availability of the vaccines, costs, logistics, side effects, and patient acceptability.

## Introduction

In December 2019, a novel severe acute respiratory syndrome coronavirus 2 (SARS-CoV-2), was first detected in Wuhan, China^1^. It causes highly infectious Coronavirus Disease 2019 (COVID-19) to spread worldwide and became a global pandemic. Despite numerous global efforts to mitigate the pandemic for almost two years, the SARS-CoV-2 continues to spread, disrupting life's routine, causing very high morbidity (above 225 million confirmed cases) and mortality (more than four and half million deaths) worldwide as of September 15, 2021^2^.

Within a short period, it became clear that the way to deal with the current pandemic is an effective therapy for severe COVID-19 patients together with preventing SARS-Cov-2 spread through population vaccination. From the beginning of the pandemic, global efforts have been focused on developing safe and efficacious vaccines for COVID-19 prevention. Until recently, vaccine development was considered a long and complicated process, lasting for decades before the product has been approved for clinical use^3^. Shortly after the start of the SARS-Cov-2 outbreak, scientists began racing to develop an effective and safe vaccine against SARS-CoV-2, based on new and old vaccines technologies^4^.

Within less than two years period, there are more than 300 vaccine candidates globally, 117 vaccines in different clinical stages of development, including 30 of them in phase 3^5^. As of mid-2021, seven COVID-19 vaccines have received emergency use authorization (EUA) in different countries, including United States (US), European Union (EU), United Kingdom (UK). These emergency authorizations of use are summarized in the World Health Organization (WHO) Emergency Use Listing: Pfizer/BioNTech (US, EU, UK, WHO), Moderna (US, EU, UK), AstraZeneca (EU, UK), Janssen (US, EU), and Gamaleya (Russian Ministry of Health), Sinopharm and Sinovac (National Medical Products Administration (NMPA), China)^5^.

The vaccines with EUA use various vaccine technologies, including mRNA^6,7^, virus vector^8–10^, and adjuvanted recombinant protein nanoparticles^11^. Each technology has its advantages and limitations^12^.

mRNA-1273^7^ and BNT162b2^6^ are the newest generations of mRNA vaccines. mRNA vaccines do not contain the antigen itself but deliver the genetic information for the antigen, and vaccinated individual synthesizes antigens in the host cells^13^. In this technology, all components are produced via chemical synthesis, which allows fast-track development in the event of a pandemic. The advantages associated with mRNA vaccines include high efficacy and relatively low severity of side effects. Before the current pandemic, mRNA vaccine technology seems promising in several diseases such as cytomegalovirus and Zika virus^14^, however, mRNA vaccines were not licensed for human use before the SARS-Cov-2 pandemic^15^. Thus, there are relatively short-term efficacy and safety data of COVID-19 mRNA vaccines, including recently published short-term real-world studies^6,7,16–20^. NVX-CoV2373 is an adjuvanted recombinant protein vaccine that contains Matrix-M1 adjuvant and a recombinant full-length wild-type SARS-CoV2 spike glycoprotein^21^. The same technology platform was used in the recently EU-approved Janssen Ebola vaccine^22^. ChAdOx1, Ad26CoV2.S, and Gam-COVID-Vac are viral vector-based vaccines^8–10^. The technology uses antigen cloned into a viral vector that cannot reproduce. The viral vector imitates the viral infection disease state and can produce more robust cellular immune responses compared to the recombinant protein vaccine. Adenoviral vector vaccines' safety has been extensively studied, and adenoviral vector-based therapeutic drugs are used in clinical practice^23^. In parallel with new technologies, recently published RCT reported the efficacy of three new whole-virus inactivated vaccines^24,25^.

For most new SARS-CoV-2 vaccines the efficacy data are based on the results of single phase 3 RCT, together with recently published real-world data for some of them^6–11,17,19,24,25^. Widespread vaccination programs have commenced in several countries, while the long-term effectiveness of COVID-19 vaccines is lacking. Recently published meta-analysis of eight COVID-19 vaccines, that have published the data of phase 3 randomized controlled trials (RCTs), reported excellent efficacy (pooled Risk Ratio (RR) to prevent symptomatic disease of 0.17; 95% Confidence Interval (CI): 0.09–0.32)^26^. While all new COVID-19 vaccines were found to be very effective to prevent symptomatic disease as compared to control, no study compared the efficacy between different vaccines.

The conventional meta-analysis approach can only compare two interventions at a time. Using the network methods enables the evaluation of multiple treatments in a single analysis. In the absence of a trial that directly compared two different treatments, an indirect comparison can be performed. Indirect evidence refers to the evidence obtained through a common comparator^27^. Network meta-analysis published on March 2021 included data about four COVID-19 vaccines and provided the following rank of effectiveness: BNT162b2 **≈** mRNA-1273 > Gam-COVID-Vac >  > ChAdOx1^28^.

We aimed to integrate updated published data from phase 3 RCTs about different COVID-19 vaccines and provide an indirect comparison between vaccines' clinical efficacy to prevent symptomatic and severe disease, using network meta-analysis. Our results may provide additional evidence-based information to help choose the best policy to achieve the most significant public health benefit.

## Methods

### Data sources and search strategy

We performed a comprehensive database search which included PubMed/Medline, Embase, including Mesh/Emtree terms search, Clinical Trials Registry Clinicaltrials.gov, and The Cochrane Library using the following keywords: COVID-19, severe acute respiratory syndrome coronavirus, Coronaviridae Infections, coronavirus, sudden acute respiratory syndrome, vaccines, vaccine, randomized controlled trial, controlled clinical trial, clinical trial, phase II/III, phase III. The search strategies incorporated index terms (Mesh) and text words for the search concepts. The search words are detailed in online-only supplements. Databases were searched up to August 30, 2021, without language or date restrictions.

The primary outcomes were the clinical efficacy of the vaccine against symptomatic laboratory-confirmed COVID-19. Secondary outcomes were the efficacy to prevent severe COVID-19 infection and vaccine efficacy among the elderly.

The systematic review and network meta-analysis were performed following *Preferred Reporting Items for Systematic Reviews and Meta-Analysis* (PRISMA) 2020 framework guidelines^29^. The protocol was registered in *the International Prospective Register of Systematic Reviews* (PROSPERO) on February 5, 2021 (CRD42021235364).

### Inclusion and exclusion criteria

We included published phase 3 RCTs to evaluate the vaccine's efficacy to prevent symptomatic COVID-19. The following publications were excluded from analysis: phase 1 and phase 2 RCTs, non-randomized trials, observational studies, duplicated reports, pharmacokinetic studies in healthy adults, reviews, expert opinion, editorials, letters to the editor, and comments.

### Data extraction

One reviewer (V.R.) identified the studies. Two reviewers (V.R., B.H.R.) independently examined the list of titles, the abstracts, and finally, the full-text articles for eligibility using the Rayyan web software for systematic reviews^30^. Disagreements were resolved through consensus.

### Data collection

The following data were extracted by two independent reviewers: study details (identifier, study design, geographical location, study period, publication year, length of follow up), participant details (number of participants, study population, age and gender, co-morbidities, SARS-Cov-2 variants), intervention details (vaccine name, vaccine platform, vaccine regimen), details about efficacy outcomes: number of cases of symptomatic disease, number of cases of severe disease, number of cases of symptomatic disease in participants above the age of 60 years (raw data). Disagreements between reviewers were resolved through consensus.

### Quality assessment and risk of bias

The risk of bias of the randomized control trials was assessed by two independent reviewers using the Cochrane tool for assessing the risk of bias for randomized control trials (RCT)^31^.

### Statistical analysis

We implemented a network meta-analysis according to PRISMA-NMA 2015^32^. To investigate the differences in efficacy between various vaccines, we performed a pairwise network meta-analysis, using a random-effects model^33–36^. In the absence of trials that directly compared two different vaccines, only indirect comparisons have been performed. The network incorporated raw data of vaccine efficacy compared to control from each included study. RRs and 95% CIs for indirect comparisons between different vaccines regarding their relative efficacy was calculated using the pairwise method.

Vaccine efficacy was ranked using P-scores derived from network point estimates. The P-score is a frequentist equivalent to the Bayesian network surface under the cumulative ranking curve. The P-score of intervention can be interpreted as the mean extent of certainty that one intervention is better than another intervention, and can be used to rank an intervention within a range of interventions, measured on a scale from 0 (worst) to 1 (best)^37^.

To compare vaccines efficacy to prevent severe disease, we incorporated raw data of severe cases among vaccinated and control groups, as reported in each study. RRs and 95% CIs for indirect comparisons between different vaccines regarding their relative efficacy was calculated using the pairwise method. Vaccine’s efficacy to prevent severe disease was ranked using P-scores derived from network point estimates.

We applied pairwise network meta-analysis, using a random-effects model to compare vaccines’ efficacy to prevent symptomatic disease among the elderly. The network incorporated raw data of vaccine efficacy compared to control in patients above 60 years old from each included study. Vaccine’s efficacy to prevent symptomatic disease among the elderly was ranked using P-scores derived from network point estimates.

Analysis was performed using R Version 3.4.3 and the “netmeta” package Version 0.9–8^38^.

## Results

We identified eight phase-3 RCTs that reported primary or preliminary CODIV-19 vaccine efficacy, with contributory data from nine publications^6–11,24,25,39^.

The search and selection processes are illustrated in eFigure 1. The characteristics of included studies are summarized in Table 1. Data from above two hundred thousand participants are included in our network meta-analysis. Of whom 114,247 (52%) received the intervention (active COVID-19 vaccine), most of the participants (above 70%) are adults below the age of 60 years. The average number of participants per trial was 24,252 (± 9,877). A total of 1,419 cases of the primary outcome were reported in the included studies (eTable 1).

**Table 1:** Characteristics of included studies.

Author	Trial period	Geographical location	Intervention	Vaccine type	Pharma	Regiment	# participants	Age (mean, range)	Gender (male, %)
Polack FP^6^	July 27—Nov 14, 2020	US, Argentina, Brazil, South Africa, Germany, Turkey	BNT162b2	mRNA	Pfizer/ BioNTech	2 doses, 21 days apart	37,706	52 (16–91)	50.6
Baden LR^7^	July 27—Oct 23, 2020	US	mRNA-1273	mRNA	Moderna	2 doses, 28 days apart	30,351	51.4 (18–95)	52.7
Voysey M^8^	April 23—Nov 4, 2020	UK, Brazil	ChAdOx12	Viral Vector including S-protein DNA	Astra Zeneca/ Oxford	2 doses, 4–12 weeks apart	11,636	18 +	39.5
Logunov DY^10^	Sept 7—Nov 24, 2020	Russia	Gam-COVID-Vac	Viral Vector including S-protein cDNA	Gamaleya NRCEM	2 doses, 21 days apart	19,866	45 (SD 12)	61.2
Heath PT ^11^	Sep 28 – Nov 28, 2020	UK	NVX-CoV23730	Recombinant S-protein	Novavax	2 doses 21 days apart	14,039	56 (18–84)	51.6
Sadoff J^9^	- January 22, 2021	US, South Africa, Latin America	Ad26.COV2.S	Viral vector expressing S protein	Janssen/ Johnsen & Johnsen	1 dose	38,484	52.0 (18–100)	54.9
Kaabi NA^24^	-December 20, 2020	United Arab Emirates, Bahrain	WIV04, HB02	Inactivated viruse strains	Sinopharm -Beijing	2 doses 21 days apart	38,206	36.1 (± 9.3)	84.4
Tanriover MD ^25^	Sept 15, 2020, and Jan 6, 2021	Turkey	CoronaVac	Inactivated whole-virion	Sinovac Life Sciences	2 doses 21 days apart	10,214	18–59	57.8

### Indirect comparison

#### Symptomatic disease

**Figure 1:**
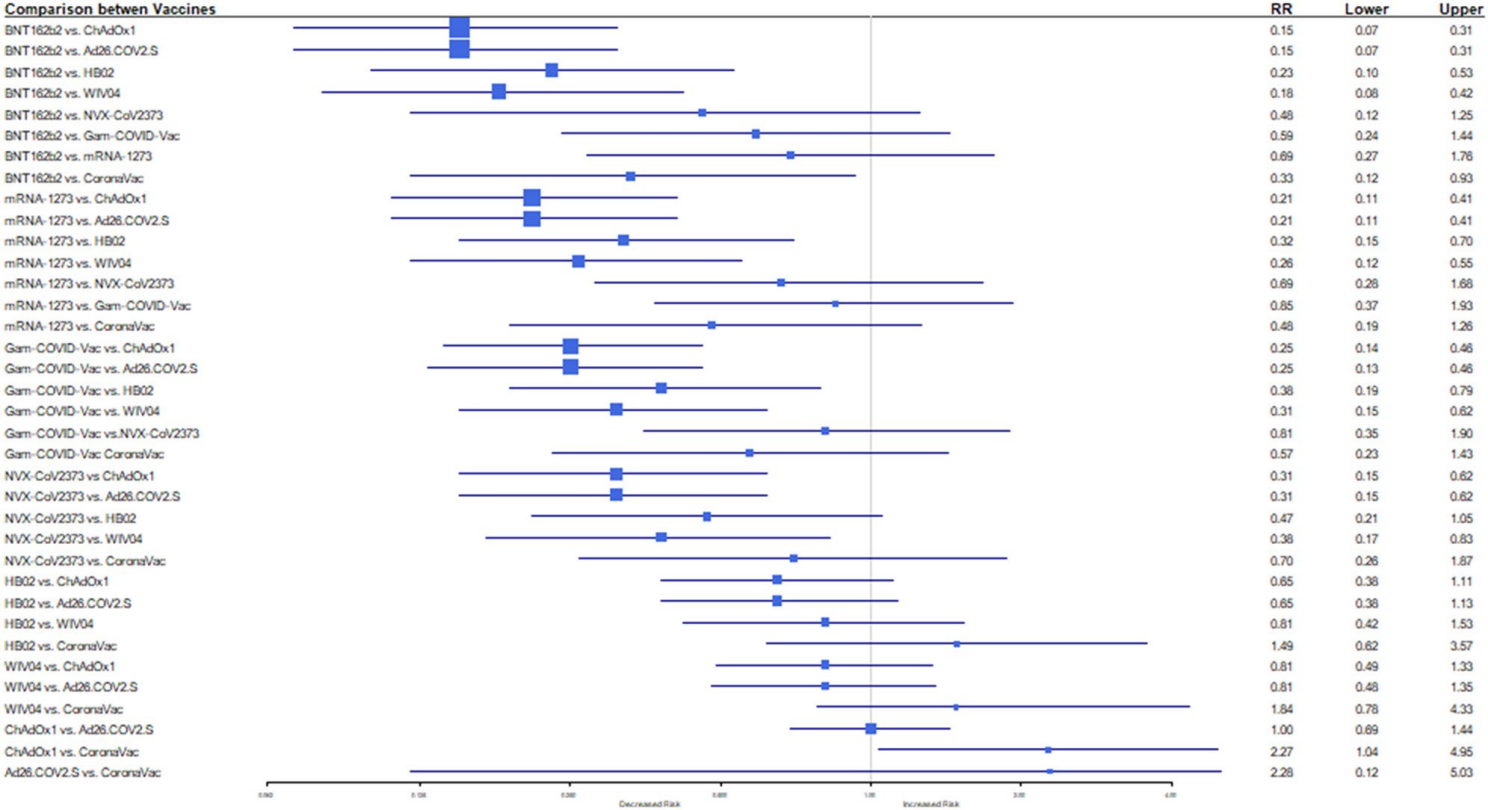
Results of random-effects network meta-analysis for efficacy to prevent symptomatic COVID-19: Risk Ratio (RR) for indirect comparison between the vaccines or vaccine vs. placebo, and 95% confidence intervals (Seven studies included).

Our search revealed information about efficacy of nine new vaccines to prevent symptomatic COVID-19 (Table 1). When the indirect comparison between the vaccines was performed, BNT162b2 and mRNA-1273 vaccines were ranked with the highest probability of efficacy against symptomatic COVID-19 (P-score: 0.952, 0.843, respectively), followed by Gam-COVID-Vac (P-score 0.782), NVX-CoV23730 (P-score 0.700), CoronaVac (P-score 0.570), BN02 (P-score 0.428), WIV04 (P-score 0.327), ChAdOx1 (P-score 0.199), and Ad26.COV2.S (P-score 0. 0.198) (Table 2). BNT162b2, mRNA-1273, Gam-COVID-Vac, and NVX-CoV23730 vaccines were statistically significantly associated with a decreased risk for symptomatic COVID-19 (Fig. 1). Comparison of BNT162b2: RR 0.15, 95% CI: 0.07–0.31 vs. ChAdOx1 and Ad26.COV2.S; 0.23 (0.10–0.53) vs. HB02; 0.18 (0.08–0.42) vs. WIV04. Comparison of mRNA-1273: 0.21 (0.11–0.41) vs. ChAdOx1 and Ad26.COV2.S; 0.32 (0.15–0.70) vs. HB02; 0.26 (0.12–0.55) vs. WIV04. Comparison for Gam-COVID-Vac: 0.25 (0.14–0.46) vs. ChAdOx1 and Ad26.COV2.S; 0.38 (0.19–0.79) vs. HB02; 0.31 (0.15–0.62) vs. WIV04. Comparison for NVX-CoV23730: 0.31 (0.15–0.62) vs. ChAdOx1, and Ad26.COV2.S, and 0.38 (0.17–0.83) vs. WIV04.

**Table 2:** P-Score ranking vaccines’ efficacy to prevent COVID-19.

Vaccine	P-Score ranking^a^		
	Symptomatic disease	Severe disease	Symptomatic disease in elderly^b^
BNT162b2	0.953	0.499	0.815
mRNA-1273	0.844	0.816	0.573
Gam-COVID-Vac	0.782	0.899	0.722
NVX-CoV2373	0.701	0.531	0.623
CoronaVac	0.570		
HB02	0.428	0.384	
WIV04	0.327	0.384	
Ad26.COV2.S	0.198	0.434	0.262
ChAdOx1	0.199		

^a^P-score represents the probability of each intervention is being better than all competing interventions, derived from network point estimates and standard errors.

^b^Subjects above 60 years.

#### Age 60 and above

**Figure 2:**
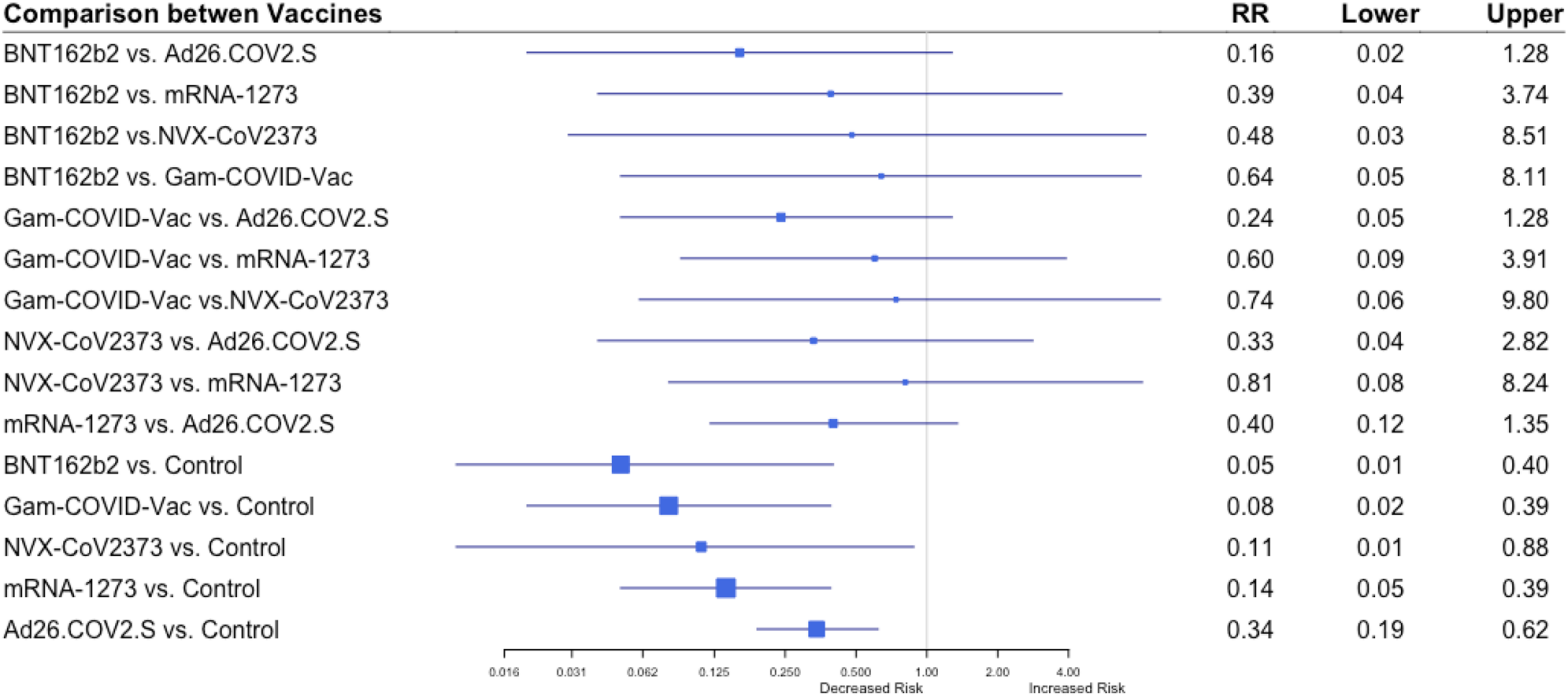
Results of random-effects network meta-analysis for efficacy to prevent symptomatic COVID-19 in subjects ≥ 60 years old: Risk Ratio (RR) for indirect comparison between the vaccines or vaccine vs. placebo, and 95% confidence intervals (Four studies included).

Five studies reported vaccines' efficacy to prevent symptomatic disease among the older population (60 years and above) =^6,7,9–11^. The network incorporated 128 cases of symptomatic disease among patients above age 60 in vaccine and control groups, as reported in each study (eTable 1). When the indirect comparison between the vaccines was performed, BNT162b2 was ranked with the highest efficacy against symptomatic COVID-19 (P-score 0.815), followed by Gam-COVID-Vac (P-score 0.722), NVX-CoV23730 (P-score 0.623), mRNA-1273 (P-score 0.573), and Ad26.COV2.S (P-score 0.263) (Table 2). However, no vaccine was statistically significantly associated with a decreased risk compared to other vaccines (Fig. 2).

#### Development of severe disease

**Figure 3:**
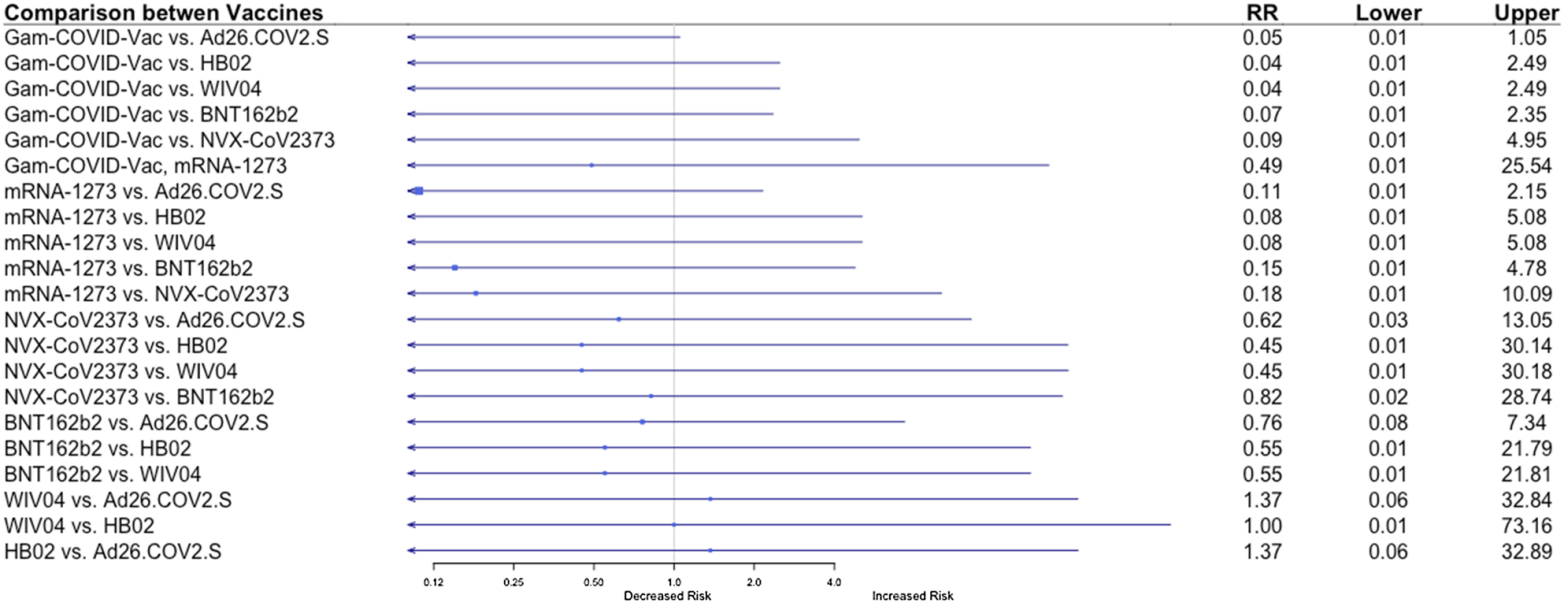
Results of random-effects network meta-analysis for efficacy to prevent severe COVID-19: Risk Ratio (RR) for indirect comparison between the vaccines or vaccine vs. placebo, and 95% confidence intervals (Five studies included).

Additionally, we evaluated the efficacy of the vaccines to prevent clinically significant severe COVID-19. The data of severe disease were available from five studies, a total of 107 cases of severe disease (eTable 1)^6,7,9–11,24^. eTable 2 summaraizes sever COVID-19 definitions, as defind in the inclided studies. When the indirect comparison between the seven vaccines was performed, Gam-COVID-Vac and mRNA-1273 vaccines were ranked with the highest efficacy to prevent a severe COVID-19 (P-scores 0.899 and 0.816, respectively), followed by NVX-CoV23730 (P-score 0.531), BNT162b2 (P-score 0.500), Ad26.COV2.S (P-score 0.34), WIV04 and HB02 (P-score 0.384) (Table 2). However, no vaccine was statistically significantly associated with a decreased risk compared to other vaccines, although there was a trend present with mRNA-1273 and Gam-COVID-Vac vaccines compared to the other vaccines for a lower risk for severe disease (Fig. 3).

### Risk of bias

The risk of bias was evaluated for all published studies. It was classified as having some concerns for four studies^6–8,11^ and it was deemed moderate for other studies^9,10,24,25^ (eFigure 2).

## Discussion

Over the last year, we have witnessed the development and clinical introduction of very effective COVID-19 vaccines, based on results from phase 3 RCTs. The two-dose regimen of BNT162b2 and mRNA-1273 mRNA, two vaccines based on new mRNA technology, presented extremely effective protection against COVID-19 (95% and 94.1%, respectively)^6,7^. Different regimens of viral-vector vaccines expressing SARC-CoV-2 S protein: Gam-COVID-Vac, Ad26.COV2.S, and ChAdOx1, were highly effective to protect against symptomatic COVID-19 (91.6%, 66.9%, and 66.7%, respectively)^8–10^. A two-dose regimen of the NVX-CoV2373, recombinant S-protein vaccine, administered to adult participants conferred 89.7% protection against SARS-CoV-2 infection^11^. Recently published results of three inactivated vaccines developed from different SARS-CoV-2 strains reported high efficacy for preventing COVID-19 symptomatic disease (83.5% CoronaVac, 78.1% HB02, and 72.8% WIV04)^24,25^. Combined data from phase 3 RCTs reported excellent efficacy of eight COVID-19 vaccines to prevent symptomatic disease as compared to control (RR 0.17; 95% CI 0.09–0.32)^26^.

The first network meta-analysis to compare the clinical efficacy of new COVID-19 vaccines was published on March 2021 and included four interventions: BNT162b2, mRNA-1273, Gam-COVID-Vac, and ChAdOx1^28^. The current research is the most comprehensive network meta-analysis to compare the efficacy of nine new COVID-19 vaccines to prevent symptomatic and severe disease in the adult population.

### Symptomatic disease

In our indirect comparison, the mRNA vaccines: BNT162b2 and mRNA-1273 were associated with the highest decrease in the relative risk for symptomatic COVID-19 compared to the other vaccines. BNT162b2 vaccine was associated with an 85% decreased relative risk of symptomatic disease than ChAdOx1 and Ad26.COV2.S (RR 0.15, 95% CI 0.07–0.31 and RR 0.15, 95% CI 0.07–0.31, respectively). the mRNA-1273 vaccine was 79% more effective in preventing symptomatic COVID-19 than ChAdOx1 and Ad26.COV2.S (RR 0.21, 95% CI 0.11–0.41 and RR 0.21, 95% CI 0.11–0.41, respectively) (Fig. 1). Ranking BNT162b2 and mRNA-1273 vaccines as best interventions over other competing vaccines to prevent symptomatic disease (P-score 0.95 and 0.84, respectively) (Table 2). Our results are consistent with previously published data, provided the following rank of effectiveness: BNT162b2 ≈ mRNA-1273 > Gam-COVID-Vac >  > ChAdOx1^28^.

We did not find any statistically significant difference between the vaccines’ efficacy to prevent symptomatic disease among the elderly.

### Development of severe disease

Among seven vaccines included in the analysis, Gam-COVID-Vac and mRNA-1273 vaccines were ranked with the highest probability to prevent a severe COVID-19 (P-scores 0.899 and 0.816, respectively) (Table 2). However, we did not find a statistically significant difference between the efficacy of Ad26.COV2.S vaccine to prevent severe COVID-19 as compared to Gam-COVID-Vac and mRNA-1273 vaccines (RR 0.11, 95% CI 0.01–2.15 for mRNA-1273 vs. Ad26.COV2.S and RR 0.05, 95% CI 0.01–1.05 for Gam-COVID-Vac vs. Ad26.COV2.S) (Fig. 2). We infer that there was not enough statistical power to compare vaccines’ efficacy to prevent severe COVID-19, as an absolute number of events was low (107 cases of severe COVID-19) (eTable 1).

The Ad26.COV2.S, ChAdOx1, and Gam-COVID-Vac are DNA vaccines encoding the SARS-CoV-2 spike (S) protein^40^. In our analysis, the Gam-COVID-Vac vaccine was more effective in preventing symptomatic COVID-19 as compared to Ad26.COV2.S and ChAdOx1 vaccines (RR 0.25, 95% CI 0.14–0.46 and RR 0.25, 95% CI 0.14–0.46, respectively) (Fig. 1). One possible explanation for the reduced efficacy of Ad26.COV2.S vaccine is a single-dose regimen compared to the two-dose regimen of Gam-COVID-Vac. A study is evaluating a two-dose administration of Ad26.COV2.S vaccine began participant recruitment during November 2021^41^. Also, higher efficacy of Gam-COVID-Vac as indirectly compared to ChAdOx1 vaccine may be explained by two different vectors’ technology used in former. Using heterologous viral vectors for each dose allows the minimization of host immune responses against the vector components^42^. Three inactivated vaccines (HB02, WIV04, and CoronaVac) were developed from different SARS-CoV-2 strains isolated in China^24,25^. All three vaccines had comparable efficacy in preventing symptomatic COVID-19 (RR 0.81, 95% CI 0.43–1.54 for HB02 vs. VIW04, RR 1.49, 95% CI 0.62–3.57 for HB02 vs. CoronaVac, and RR 0.81, 95% CI 0.49–1.35 for VIW04 vs. CoronaVac) (Fig. 1).

### Implications

Based on the indirect comparison method, the BNT162b2 and mRNA-1273 were associated with the highest efficacy in preventing symptomatic COVID-19. Our finding may have importance when deciding which vaccine to use, although this is not the only consideration that should be considered. Availability of the vaccines, costs, logistics, side effects, and patient acceptability, amongst others, are also factors to be considered.

### Strengths and limitations

To the best of our knowledge, this is the most comprehensive network meta-analysis to compare the efficacy of nine new COVID-19 vaccines to prevent symptomatic and severe disease in the adult population. Previously published network meta-analysis reported indirect comparisons across fore COVID-19 vaccines^28^.

The results of our indirect comparison between the new vaccines showed that mRNA vaccines (BNT162b2 and mRNA-1273) were associated with a more significant decrease in the risk for symptomatic COVID-19 compared to other vaccines. We also found a trend to increased the efficacy of mRNA vaccines to prevent severe COVID-19. However, the results did not reach statistical significance because of the relatively low rate of severe disease.

However, our indirect comparison has several limitations.

Firstly, our network meta-analysis includes one study for each intervention arm. In addition, the results of the two studies are not peer-reviewed, while reported data originated from press releases and reports submitted to FDA^43,44^. There are several significant differences between studies' protocols, which may be partially responsible for the differences between the vaccine efficacies. As mentioned above, Ad26.COV2.S efficacy is based on a single-dose regimen, while other vaccines were administered as a two-dose regimen, including ChAdOx1 (AZD1222) vaccine, whose protocol was adapted to a two-dose regimen after the study had been started^45^. Moreover, vaccines were examined under non-equivalent conditions, including countries with unlike socio-economic conditions and various stages of COVID-19 outbreak, different seasons, and different SARS-CoV-2 variants. All mentioned above may influence vaccines' efficacy. Recently published data support that the B.1.1.17 variant, known as the UK strain, is susceptible to the immunity induced bytheBNT162b2 and mRNA-1273 vaccines^46,47^. However, the B.1.351 variant, primarily identified in South Africa, is less susceptible to mRNA-1273 vaccine-induced neutralizing antibodies^47^. It remains to determine if the reduction in antibody susceptibility will be associated with decreased vaccine effectiveness. There is also a high probability that the virus will acquire new mutations that will change its susceptibility to vaccines, and some vaccines might be influenced more than others. As a result, the efficacy of the different vaccines is expected to be affected. Besides, the current data on vaccine efficacy is based on short-term data, so we could not compare the effectiveness and immunity duration of different vaccines. Presently, it is not known which vaccine will induce longer immune responses. Also, as seen with other vaccines, booster doses may be required every few years to maintain immunity.

Secondly, our meta-analysis compares the efficacy of the studied vaccines in two hundred thousand participants of phase 3 RCTs without data from observational studies. So far, millions of people have been vaccinated around the world. One study from Clalit Health Services, a large health maintenance organization in Israel, compares the efficacy of the BNT162b2 mRNA vaccine in about 600,000 vaccinated persons to that of a similar-sized group of unvaccinated controls^48^. In this study, the efficacy of the BNT162b2 mRNA vaccine was similar to that seen in the phase 3 RCT^7^.

Finally, the safety outcomes of the vaccines were beyond the aims of the current network meta-analysis. Currently, available safety results are based on short-duration follow-up, and a very low rate of severe adverse reactions has been observed in the short term. As the mRNA vaccine technology is new and it is still unclear which issues will emerge in the long term, real-world data will be needed to assess the safety of prospective vaccines.

## Conclusion

In our indirect comparison, the BNT162b2 and mRNA-1273 vaccines, which use mRNA technology, were associated with the highest efficacy in preventing symptomatic COVID-19 compared to the other vaccines. The compared vaccines were not different in efficacy to prevent severe disease. We found no difference between vaccines’ efficacy to prevent symptomatic COVID-19 among the elderly.

## Supplementary Information

Supplementary Information.
