# Supplementary material for: Comparing the clinical efficacy of COVID-19 vaccines: a systematic review and network meta-analysis
Source: Sci Rep. 2021 Nov 23;11:22777. doi: 10.1038/s41598-021-02321-z (PMC8611039; doi:10.1038/s41598-021-02321-z)
Supplement: Supplementary file 1 — Supplementary Information. [file 41598_2021_2321_MOESM1_ESM.docx]

**Supplementary material**

**Comparing the Clinical Efficacy of** **COVID-19 vaccines: A systematic review and network meta-analysis**

Victoria Rotshild, PharmD, PhD, Bruria Hirsh Raccah, PharmD, PhD, Ian Miskin, MD, Mordechai Muszkat, MD, Ilan Matok, PhD

**Words Search Strategy**

**eFigure 1. Studies selection flowchart**

**eFigure 2. Risk of bias**

**eTable 1. Additional data from included studies**

**eTable 2. Definition of Severe Disease**

**Words Search Strategy**

(('coronavirinae'/exp OR 'coronaviridae infection'/exp) OR (coronavirus:ti,ab,kw OR 'corona virus':ti,ab,kw OR cov:ti,ab,kw OR ncov:ti,ab,kw OR 'covid19':ti,ab,kw OR 'covid 19':ti,ab,kw OR sars:ti,ab,kw OR mers:ti,ab,kw OR 'severe acute respiratory syndrome':ti,ab,kw OR 'middle east respiratory syndrome':ti,ab,kw OR 2019ncov:ti,ab,kw OR cov2:ti,ab,kw OR 'sudden acute respiratory syndrome':ti,ab,kw OR oc43:ti,ab,kw OR 229e:ti,ab,kw OR nl63:ti,ab,kw OR huk1:ti,ab,kw OR 'sars cov 2':ti,ab,kw OR 'mers cov':ti,ab,kw)) AND ('vaccine'/exp OR 'vaccin':ti,ab,kw OR 'vaccination':ti,ab,kw OR 'vaccine':ti,ab,kw OR 'vaccines':ti,ab,kw OR 'covid 19 vaccine'/exp) AND ('phase 2/3':ti,ab,kw OR 'phase ii/iii':ti,ab,kw OR 'phase 3 clinical trial'/exp OR 'phase 3 clinical study':ti,ab,kw OR 'phase 3 clinical trial':ti,ab,kw OR 'phase 3 study':ti,ab,kw OR 'phase 3 trial':ti,ab,kw OR 'phase iii clinical study':ti,ab,kw OR 'phase iii clinical trial':ti,ab,kw OR 'phase iii study':ti,ab,kw OR 'phase iii trial':ti,ab,kw OR 'phase 3 clinical trial topic'/exp) AND ('clinical trial'/de OR 'randomized controlled trial'/de OR 'randomization'/de OR 'single blind procedure'/de OR 'double blind procedure'/de OR 'crossover procedure'/de OR 'placebo'/de OR 'prospective study'/de OR ('randomi?ed controlled' NEXT/1 trial*) OR rct OR 'randomly allocated' OR 'allocated randomly' OR 'random allocation' OR (allocated NEAR/2 random) OR (single NEXT/1 blind*) OR (double NEXT/1 blind*) OR ((treble OR triple) NEAR/1 blind*) OR placebo*) AND ([article]/lim OR [article in press]/lim) AND [2-2-2021]/sd NOT [12-3-2021]/sd AND [2021-2021]/py

**References**

1. Polack, F. P. *et al.* Safety and Efficacy of the BNT162b2 mRNA Covid-19 Vaccine. *N. Engl. J. Med.* **383**, 2603–2615 (2020).

2. Baden, L. R. *et al.* Efficacy and Safety of the mRNA-1273 SARS-CoV-2 Vaccine. *N. Engl. J. Med.* **384**, 403–416 (2021).

3. Voysey, M. *et al.* Safety and efficacy of the ChAdOx1 nCoV-19 vaccine (AZD1222) against SARS-CoV-2: an interim analysis of four randomised controlled trials in Brazil, South Africa, and the UK. *www.thelancet.com* **397**, 2021 (2020).

4. Logunov, D. Y. *et al.* Articles Safety and efficacy of an rAd26 and rAd5 vector-based heterologous prime-boost COVID-19 vaccine: an interim analysis of a randomised controlled phase 3 trial in Russia. (2021) doi:10.1016/S0140-6736(21)00234-8.

5. Heath, P. T. *et al.* Safety and Efficacy of NVX-CoV2373 Covid-19 Vaccine. *N. Engl. J. Med.* (2021) doi:10.1056/NEJMOA2107659.

6. Sadoff, J. *et al.* Safety and Efficacy of Single-Dose Ad26.COV2.S Vaccine against Covid-19. *N. Engl. J. Med.* (2021) doi:10.1056/NEJMoa2101544.

7. Al Kaabi, N. *et al.* Effect of 2 Inactivated SARS-CoV-2 Vaccines on Symptomatic COVID-19 Infection in Adults: A Randomized Clinical Trial. *JAMA* (2021) doi:10.1001/jama.2021.8565.

8. Tanriover, M. D. *et al.* Efficacy and safety of an inactivated whole-virion SARS-CoV-2 vaccine (CoronaVac): interim results of a double-blind, randomised, placebo-controlled, phase 3 trial in Turkey. *Lancet* **398**, 213–222 (2021).


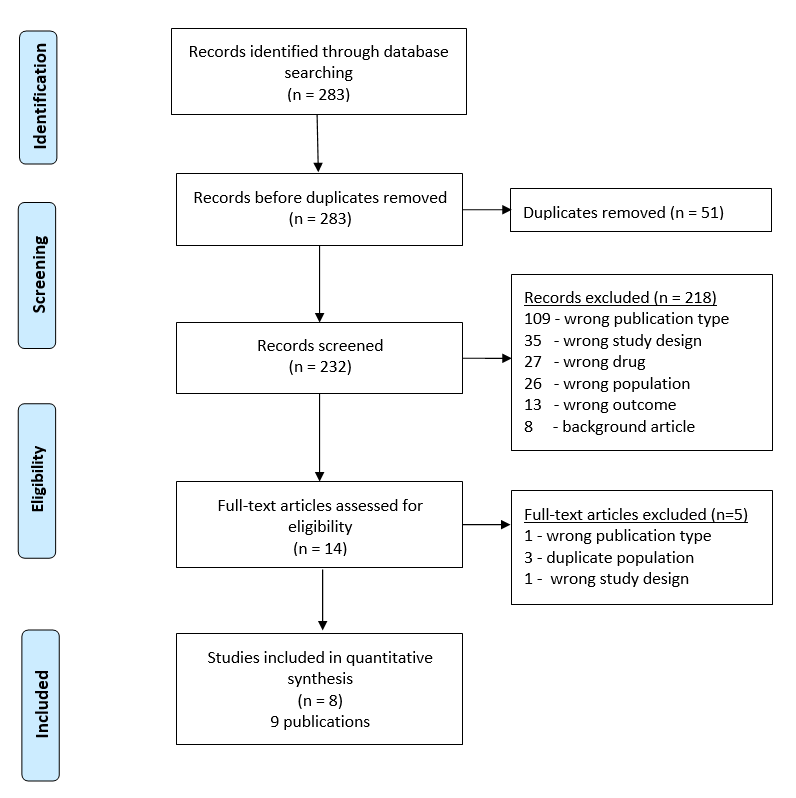


**eFigure 1. Studies selection flowchart**


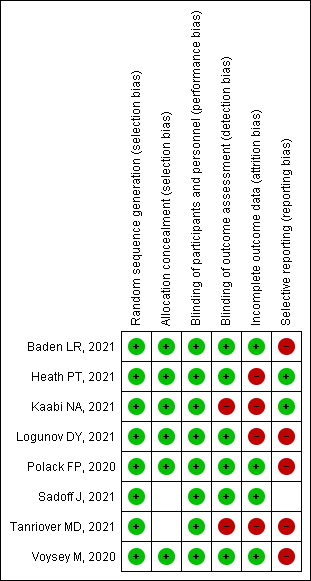


**eFigure 2. Risk of bias**

**eTable 1. Additional data from included studies**

| **Author** | **Intervention** | **Time of Primary Outcome** | **Symptomatic COVID-19** | | **Severe COVID-19** | | **Symptomatic COVID-19**  **(above 60 years old)** | |
| --- | --- | --- | --- | --- | --- | --- | --- | --- |
|  |  |  | **Intervention** (cases/total) | **Control**  (cases/total) | **Intervention** (cases/total) | **Control**  (cases/total) | **Intervention** (cases/total) | **Control**  (cases/total) |
| Polack FP [1] | BNT162b2 | At least 7 days after the 2^nd^ dose | 8/18198 | 162/18325 | 1/21314 | 9/21259 | 1/3848 | 19/3880 |
| Baden LR [2] | mRNA-1273 | At least 14 days after the 2^nd^ dose | 11/14134 | 185/17073 | 0/15170 | 30/15181 | 4/3583 | 29/3552 |
| Voysey M [3] | ChAdOx1 | At least 14 days after the 2^nd^ dose | 84/8597 | 248/8580 |  |  |  |  |
| Logunov DY [4] | Gam-COVID-Vac | At least 21 days after the 1^st^ dose | 16/14 964 | 62/4902 | 0/14964 | 20/4902 | 2/1611 | 8/533 |
| Heath PT [5] | NVX-CoV23730 | At least 7 days after the 2^nd^ dose | 10/7020 | 96/7019 | 0/7020 | 5/7019 | 1/1953 | 9/1957 |
| Sadoff J [6] | Ad26.COV2.S | At least 28 days after the dose | 66/19306 | 193/19178 | 5/19306 | 34/19178 | 14/6689 | 43/6651 |
| Kaabi NA [7] | Inactivated WIV04 | At least 14 days after the 2^nd^ dose | 26/12743 | 95/12737 | 0/12743 | 2/12737 |  |  |
| Kaabi NA [7] | Inactivated HB02 | At least 14 days after the 2^nd^ dose | 21/12726 | 95/12737 | 0/12726 | 2/12737 |  |  |
| Tanriover MD [8] | CoronaVac | At least 14 days after the 2^nd^ dose | 9/6559 | 32/3470 |  |  |  |  |

**eTable 2. Definition of Severe Disease**

| **Author** | **Sever COVID-19 Infection definition** |
| --- | --- |
| Polack FP[1] | Confirmed Covid-19 with one of the following additional features: clinical signs at rest that are indicative of severe systemic illness; respiratory failure; evidence of shock; significant acute renal, hepatic, or neurologic dysfunction; admission to an in- tensive care unit; or death (FDA definition) |
| Baden LR [2] | Confirmed Covid-19 with one of the following criteria: respiratory rate of 30 or more breaths per minute; heart rate at or exceeding 125 beats per minute; oxygen saturation at 93% or less while the participant was breathing ambient air at sea level or a ratio of the partial pressure of oxygen to the fraction of inspired oxygen below 300 mm Hg; respiratory failure; acute respiratory distress syn- drome; evidence of shock (systolic blood pressure <90 mm Hg, diastolic blood pressure <60 mm Hg, or a need for vasopressors); clinically significant acute renal, hepatic, or neurologic dysfunction; admission to an intensive care unit; or death. |
| Voysey M [3] | Hospitalizations |
| Logunov DY [4] | Moderate course: Fever over 38.5o C; Respiratory rate (RR) more than 22/min; Shortness of breath during physical exertion; Pneumonia (confirmed by computed tomography [CT] of the lungs); Oxygen saturation level < 95%; C-reactive protein (CRP) of blood serum more than 10 mg/l  Sever course: RR more than 30/min; Oxygen saturation level ≤ 93%; Oxygen partial pressure/inspiratory oxygen fraction ≤ 300 mmHg; Progression of changes in the lungs according to X-ray, CT, ultrasonography (U/S) (increase in the volume of changes in the lungs by more than 50% after 24-48 hours); Decreased level of consciousness, agitation; Unstable hemodynamics (systolic blood pressure less than 90 mm Hg or diastolic blood pressure less than 60 mm Hg, diuresis less than 20 mL/hr); Arterial blood lactate > 2 mmol/l; More than 2 points on the Sequential Organ Failure Assessment Scale )SOFA) scale |
| Heath PT [5] | Confirmed Covid-19 with one of the following additional features: Tachypnea: ≥30 breaths per minute at rest. Resting heart rate ≥125 beats per minute, SpO2: ≤93% on room air or PaO2/FiO2 <300 mmHg, High flow oxygen (O2) therapy or non-invasive ventilation (NIV)/non-invasive positive pressure ventilation (NIPPV) (eg, continuous positive airway pressure [CPAP] or bilevel positive airway pressure [BiPAP]), Mechanical ventilation or extracorporeal membrane oxygenation (ECMO), One or more major organ system dysfunction or failure to be defined by diagnostic testing/clinical syndrome/interventions, including any of the following: o Acute respiratory failure, including acute respiratory distress syndrome (ARDS) ( severity on the basis of the FDA criteria) |
| Sadoff J [6] | Severe–critical COVID-19 cases were defined by a positive RT-PCR test for SARS-CoV-2 with one of the following features: respiratory failure; evidence of shock (systolic blood pressure 30 breaths/minute; heart rate ≥125 beats/minute; oxygen saturation of 93% or less (ambient air at sea level), or a ratio of the partial pressure of oxygen to the fraction of inspired oxygen |
| Kaabi NA [7] | Confirmed COVID-19 case meeting any one of the following criteria: Respiratory distress (RR>30 breaths/min); Oxygen saturation<93% at rest; Arterial partial pressure of oxygen (PaO2)/ fraction of inspired oxygen (FiO2)<300mmHg (1mmHg=0.133kPa); The clinical symptoms progressively worsened, and the chest imaging showed >50% obvious lesion progression within 24-48 hours. |
| Tanriover MD [8] | Hospitalizations |
